# Plasma l-arginine levels distinguish pulmonary arterial hypertension from left ventricular systolic dysfunction

**DOI:** 10.1007/s00380-017-1055-7

**Published:** 2017-10-03

**Authors:** Anna Sandqvist, Jörn Schneede, David Kylhammar, Dan Henrohn, Jakob Lundgren, Mikael Hedeland, Ulf Bondesson, Göran Rådegran, Gerhard Wikström

**Affiliations:** 1 Department of Pharmacology and Clinical Neuroscience, Clinical Pharmacology, Umeå University, 901 87 Umeå, Sweden; 2 Department of Clinical Sciences Lund, Cardiology, Lund University, Lund, Sweden; 3 The Section for Heart Failure and Valvular Disease, Skåne University Hospital, Lund, Sweden; 4 Department of Medical Sciences, Cardiology, Uppsala University, Uppsala, Sweden; 5 Department of Chemistry, National Veterinary Institute (SVA), Uppsala, Sweden; 6 Department of Medicinal Chemistry, Analytical Pharmaceutical Chemistry, Uppsala University, Uppsala, Sweden

**Keywords:** Pulmonary arterial hypertension, Left heart failure, Systolic dysfunction, l-Arginine, Dimethylarginines

## Abstract

Pulmonary arterial hypertension (PAH) is a life-threatening condition, characterized by an imbalance of vasoactive substances and remodeling of pulmonary vasculature. Nitric oxide, formed from l-arginine, is essential for homeostasis and smooth muscle cell relaxation in PAH. Our aim was to compare plasma concentrations of l-arginine, asymmetric dimethylarginine (ADMA), and symmetric dimethylarginine (SDMA) in PAH compared to left ventricular systolic dysfunction (LVSD) and healthy subjects. This was an observational, multicenter study comparing 21 patients with PAH to 14 patients with LVSD and 27 healthy subjects. Physical examinations were obtained and blood samples were collected. Plasma levels of ADMA, SDMA, l-arginine, l-ornithine, and l-citrulline were analyzed using liquid chromatography–tandem mass spectrometry (LC–MS/MS). Plasma levels of ADMA and SDMA were higher, whereas l-arginine and l-arginine/ADMA ratio were lower in PAH patients compared to healthy subjects (*p* < 0.001). Patients with PAH also had lower levels of l-arginine than patients with LVSD (*p* < 0.05). l-Arginine correlated to 6 min walking distance (6MWD) (*r*_s_ = 0.58, *p* = 0.006) and l-arginine/ADMA correlated to WHO functional class (*r*_s_ = −0.46, *p* = 0.043) in PAH. In conclusion, l-arginine levels were significantly lower in treatment naïve PAH patients compared to patients with LVSD. Furthermore, l-arginine correlated with 6MWD in PAH. l-arginine may provide useful information in differentiating PAH from LVSD.

## Introduction

**Fig. 1:**
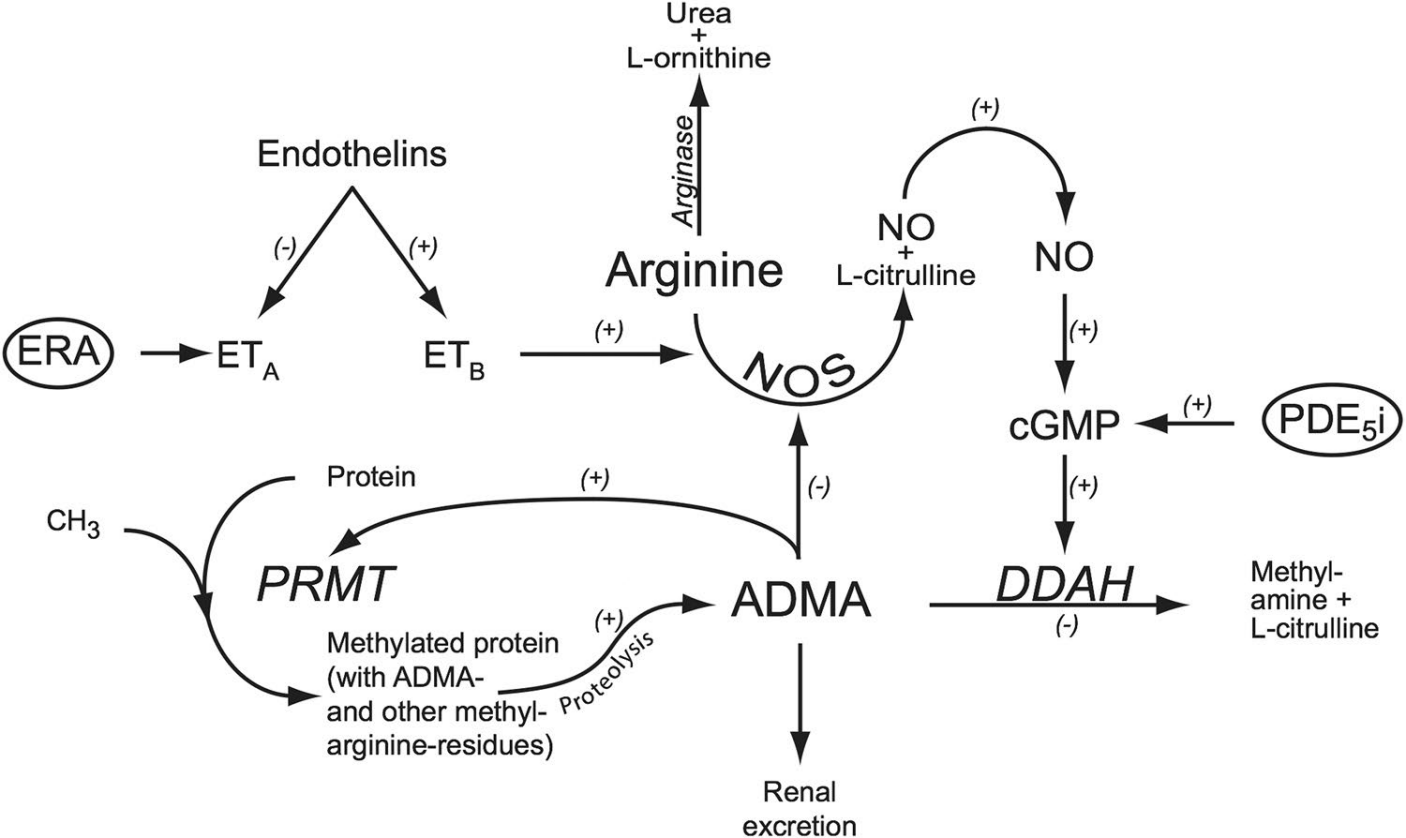
Synthesis and metabolism of ADMA through the PRMT–DDAH–ADMA pathway. Methylation of arginine residues occurs through different isoenzymes of PRMT. ADMA is an inhibitor of NOS, leading to decrease NO production. ADMA is mainly metabolized by DDAH to l-citrulline and dimethylamine and partly via urinary excretion. *ADMA* asymmetric dimethylarginine, *DDAH* dimethylarginine dimethylaminohydrolase, *ERA* endothelin receptor antagonists, *ET*_*A*/B_ endothelin receptor A/B, *PDE5i* phosphodiesterase type 5 inhibitors, *PRMT* protein arginine *N*-methyl transferases

Pulmonary arterial hypertension (PAH) is a rare disease characterized by endothelial dysfunction and pulmonary vascular remodeling, leading to elevated pulmonary vascular resistance, right ventricular heart failure, and premature death [1]. Endothelial dysfunction is characterized by increased production of vasoconstrictors, such as endothelin and thromboxane A2, and decreased production of vasodilator substances, such as prostacyclin and nitric oxide (NO). Endothelium-derived NO, specifically, is essential for homeostasis of vascular tone by activating guanylate cyclase, leading to increased formation of cGMP and subsequently smooth muscle relaxation [2]. NO is formed in the course of transformation of l-arginine to citrulline, a reaction catalyzed by the enzyme NO synthase (NOS) (Fig. 1) [3]. Moreover, l-arginine is also metabolized by arginase to l-ornithine and urea [4].

Endogenous analogues of l-arginine that selectively inhibit NOS are involved in the pathogenesis of various cardiovascular diseases [5]. There are three forms of l-arginine residues, asymmetric dimethylarginine (ADMA), symmetric dimethylarginine (SDMA), and mono-methylarginine (l-NMMA), which are formed through methylation by protein arginine *N*-methyltransferases (PRMTs) [6]. Among these, ADMA is the most potent endogenous NOS inhibitor [5]. Elevated ADMA levels can lead to decreased NO production and endothelial dysfunction [7]. Dimethylarginine dimethylaminohydrolase (DDAH) is an important regulator of NO bioavailability and cardiovascular function, by its capacity to metabolize ADMA to l-citrulline and dimethylamine [8, 9]. Oxidative stress and/or inhibition of DDAH lead to increased ADMA levels in endothelial cells and inhibit NO-mediated endothelium-dependent relaxation of blood vessels [10–12]. The lungs produce a significant amount of ADMA, suggesting that pulmonary dysregulation of ADMA metabolism could be involved in the pathogenesis of PAH [6]. Intracellular ADMA is exported into plasma and exerts negative effects on vascular homeostasis by impairing endothelial function, increasing arterial stiffness and promoting vascular inflammation [13]. Elevated plasma ADMA levels have been reported in several types of pulmonary hypertension (PH) [14–21]. Together, these findings strongly indicate an association between ADMA plasma levels and PAH/PH pathogenesis [22]. As a mediator of endothelial dysfunction and damage, ADMA could serve as a potential biomarker for PAH [18]. ADMA levels may also predict cardiovascular outcome and mortality in PAH [14].

We recently reported that plasma ADMA levels significantly decreased after a single oral dose of the PDE5-inhibitor vardenafil in patients with PH [23]. Furthermore, plasma l-arginine concentrations and the l-arginine/ADMA ratio increased over time and were correlated with changes in mean pulmonary artery pressure (mPAP), cardiac output (CO), and cardiac index (CI). The aim of the present study was to investigate baseline plasma ADMA, SDMA, and l-arginine levels in PAH patients compared to patients with left ventricular systolic dysfunction (LVSD) and healthy subjects.

## Method

### Study design and population

This was an observational and multicenter study. A total of 21 consecutive treatment naïve patients admitted for the first-time diagnostic right heart catheterization (RHC) at the Regional Centers for PAH in Uppsala or Lund between October 2010 and April 2015 were recruited. Inclusion criteria were clinical diagnosis of PAH and age above 18 years. Exclusion criteria were patients in PH group 2–5. Blood samples were taken and examined for the levels of l-arginine, ADMA, SDMA, l-ornithine, and l-citrulline. Detailed medical histories and physical examinations included age, gender, weight, body surface area (BSA), height, electrocardiography, echocardiography, 6 min walking distance (6MWD), NT-proBNP, and RHC. The 6MWD was carried out in accordance with the American Thoracic Society’s guidelines [24]. The individuals included were screened for high blood pressure, diabetes mellitus, ischemic heart disease, stroke, renal failure, and thyroid disease. For comparison, 27 healthy subjects without drug treatment and 14 patients treated for LVSD were examined for ADMA, SDMA, and l-arginine. All healthy controls were non-smokers and had no symptoms or signs of common cold at the time of recruitment.

The study was approved by the Independent Ethics Committee in Uppsala (Dnr 2010/343) and Lund (Dnr 2010/114, 2011/368, 2011/777), and conducted in accordance with the Helsinki Declaration. All patients gave their informed consent.

### Hemodynamic evaluation

All PAH patients underwent routine RHC as part of the initial diagnostic work-up. RHC was performed at the Hemodynamic Laboratory using a fiberoptic thermodilution pulmonary artery catheter, Becton–Dickinson Criticath SP5 107 HTD catheter at Uppsala University hospital, and a Swan-Ganz catheter 7.5 F (2.5 mm), Edward Lifesciences LLC, Irvine, CA, USA at Lund University hospital. The catheter was inserted through the right internal jugular vein into the pulmonary artery. Recorded hemodynamic data were: heart rate, central venous pressure (CVP), systolic pulmonary artery pressure (sPAP), mPAP, mean pulmonary artery wedge pressure (mPAWP), mean right atrial pressure (mRAP), CO, and CI. Mixed venous and arterial oxygen saturation was also measured. Pressures were registered with a Siemens^®^ system and flow was calculated with the thermodilution technique or with Fick’s principle. Pulmonary vascular resistance (PVR), systemic vascular resistance (SVR), and PVR/SVR were calculated. Systemic blood pressure was measured invasively in Uppsala and non-invasively in Lund. Experienced cardiologists performed all examinations.

### Sample preparations and analytical procedures

Blood samples were obtained from the pulmonary artery or from peripheral veins during diagnostic RHC. All blood samples were collected in EDTA tubes (BD Diagnostics, Burlington, NC, USA) and plasma was retrieved after centrifugation. Plasma was separated and stored at −70 °C in Uppsala and at −80 °C in Lund waiting for biochemical analysis.

Plasma levels of ADMA, SDMA, l-arginine, l-ornithine, and l-citrulline were analyzed with liquid chromatography–tandem mass spectrometry (LC–MS/MS) at the Swedish National Veterinary Institute in Uppsala, Sweden. Sample pretreatment was as follows. To 100 µL of plasma, 50 µL of water and 50 µL of the internal standard solution containing ^2^H_7_-ADMA, ^2^H_6_-SDMA, ^13^C_6_-arginine, 2H6-ornithine, and ^2^H_7_-citrulline were added followed by addition of 400 µL of acetonitrile/trifluoroacetic acid/propionic acid (1000/0.25/10 v/v/v). The samples were vortex-mixed for 5 min followed by centrifugation for 5 min at 10,000*g*. The supernatants were transferred vials for analysis with ultra-high-performance liquid chromatography–tandem mass spectrometry (UHPLC–MS/MS). A Waters Acquity UPLC system was coupled to a Quattro Ultima Pt tandem quadrupole mass spectrometer with an electrospray interface operating in the positive mode (Waters Corporation, Milford, MA, USA). The column was an Indra Almtakt Amino Acid (length 100 mm, I.D. 2.0 mm, particle size 3 µm) kept at 25 °C. The mobile phase consisted of (A) 100 mM ammonium formate in water and (B) 0.1% formic acid in acetonitrile. The injection volume was 10 µL. The elution was carried out as follows: isocratic at 80% A for 1 min, increase to 95% A during 1 min, constant at 95% A for 5.5 min, and decrease to 80% A during 0.1 min, constant at 80% A for 2.4 min. The flow-rate was 200 µL/min. The five analytes were quantified simultaneously in the same chromatographic run using a positive capillary voltage of 0.50 kV and a cone voltage of 40 V. The desolvation and source block temperatures were 350 and 120 °C, respectively, and the cone and desolvation gas flows were 120 and 924 L/h, respectively. The quantifications were performed in the selected reaction monitoring (SRM) mode with the collision cell filled with argon gas at a pressure of 1.9 × 10^−3^ mBar. The mass transitions used in SRM were *m/z* 203 → 46 for ADMA (collision energy 18 eV), *m/z* 210 → 77 for [^2^H_7_]-ADMA (collision energy 23 eV), *m/z* 203 → 172 for SDMA (collision energy 16 eV), *m/z* 209 → 116 for [^2^H_6_]-SDMA (19 eV), *m/z* 175 → 70 for arginine (collision energy 18 eV), *m/z* 181 → 74 for [^13^C_6_]-arginine (collision energy 18 eV), *m/z* 133 → 70 for ornithine (collision energy 13 eV), *m/z* 139 → 76 for [^13^H_6_]-ornithine (collision energy 14 eV), *m/z* 176 → 113 for citrulline (collision energy 18 eV), and *m/z* 183 → 120 for [^13^C_7_]-citrulline (collision energy 16 eV). The dwell time was 0.010 s. Stock solutions of ADMA, SDMA, arginine, ornithine, citrulline, and the internal standards were prepared in water at approximately 0.1–0.3 mg/mL. The reference standards and the internal standards were obtained from Toronto Research Chemicals (North York, ON, Canada). To check for matrix effects and to compensate for endogenous levels of the analytes in the spiked plasma, calibration samples were constructed for all three analytes in both water and in control plasma. The calibration curves were constructed using the chromatographic peak area ratio (analyte/internal standard) as a function of analyte concentration. The calibration functions were calculated by linear curve fit using a weighting factor of 1/*x* ^2^ for all three analytes. The calibration range for ADMA was 0.090–3.4 µM and the precision was in the range of 5.3–7.3%. The calibration range for SDMA was 0.38–3.0 µM and the precision was in the range of 5.8–9.3%. The calibration range for arginine was 4.5–150 µM and the precision was in the range of 3.5–6.2%. The calibration range for ornithine was 4.5–151 µM and the precision was in the range of 4.1–5.2%. The calibration range for citrulline was 4.4–150 µM and the precision was in the range of 2.5–4.8%.

### Statistical analysis

Clinical characteristics, biochemical indicators, and hemodynamic parameters were summarized by median (interquartile range) and investigated biomarkers by median (IQR) or median (range). Non-parametric tests were used due to non-normal data distributions and small sample size. Differences between two groups were evaluated with the Mann–Whitney *U* test. For testing differences between three groups (patients with PAH, LVSD and healthy subjects), the Kruskal–Wallis test was used. In cases where results of the Kruskal–Wallis test were significant, Mann–Whitney *U* tests were performed to compare the PAH patients with LVSD patients and healthy subjects, separately. Spearman’s test was used to evaluate the association between hemodynamic variables and methylarginines. Multiple linear regression analysis was used to evaluate associations between l-arginine and 6MWD. *p* values <0.05 were considered statistically significant. Statistical analyses were performed with SAS 9.3 (SAS Institute Inc, Cary, NC, USA) and IBM SPSS Statistics package v. 24.

## Results

### Demographics, biochemical, and hemodynamic characteristics

Patient characteristics and biochemical data in patients with PAH and LVSD and in healthy subjects are presented in Table 1. The PAH group consisted of 13 women (62%) and eight men (38%) with a median age of 73 (45–85) years. Four of these patients had PAH associated with connective tissue disease and 17 had idiopathic PAH. Three of the PAH patients had a history of ischemic heart disease. None of these patients had signs of left-sided heart disease during the study (PAWP = 12, 6 and 8 mmHg). Patients with PH due to left heart disease have PAWP >15 mmHg according to ESC guidelines for the diagnoses and treatment of pulmonary hypertension [1]. Hemodynamic parameters in PAH patients are shown in Table 2. The LVSD group consisted of eight women (57%) and six men (43%) with a median age of 67 (48–82) years. The majority of the LVSD patients had dilated cardiomyopathy of non-ischemic orient with a median ejection fraction of 33 (15–35) %. Based on echocardiography, no right ventricular failure was present in the patients with LVSD except in one patient. This patient underwent RHC and had an mPAP of 31, mPAWP of 16, and CO of 5.8, and was diagnosed with PH due to left heart disease. The patient is included in the result.

**Table 1:** Clinical characteristics of patients with PAH, LVSD, and healthy controls

Parameter	PAH		LVSD		Healthy subjects	
	*n*	Median (IQR)	*n*	Median (IQR)	*n*	Median (IQR)
Clinical features						
Sex (male/female)	21	8/13	14	6/8	27	8/19
Age (years)	21	73 (40)	14	67 (19)	27	61 (30–77)
Weight (kg)	21	75 (37)	13	86 (27)		N/A
BSA (m^2^)	21	1.8 (0.5)	13	1.9 (0.4)		N/A
WHO/NYHA class (II/III/IV)	21	5/15/1	14	8/6/0		N/A
6MWD (m)	18	245 (288)	12	415 (195)*		N/A
Diabetes mellitus (yes/no)	21	5/16	14	3/11	27	0/27
Hypertension (yes/no)	21	14/7	14	7/7	27	0/27
AF (yes/no)	21	4/17	14	2/12	27	0/27
Ischemic heart disease (yes/no)	21	3/18	14	1/13	27	0/27
History of stroke (yes/no)	21	1/20	14	0/14	27	0/27
Drug treatment						
Anticoagulants (yes/no)	21	11/10	14	5/9	27	0/27
Diuretics (yes/no)	21	15/6	14	13/1	27	0/27
Digitalis (yes/no)	21	7/14	14	3/11	27	0/27
Oxygen (yes/no)	21	6/15	14	0/14	27	0/27
Biochemical indicators						
NT-proBNP (ng/L)	19	2194 (2068)	14	756 (1403)*		N/A
*S*-Creatinine (µM/L)	21	98 (33)	14	97 (48)		N/A
eGFR (mL/min)	21	65.0 (30.3)	14	61.2 (38.8)		N/A

Data are presented as median (interquartile range)

*BSA* body surface area, *WHO* World Health Organization, *6MWD* six-minute walk distance, *AF* atrial fibrillation, *absolute eGFR* estimated glomerular filtration rate (Cockcroft Gault formula)

* *p* value for comparison between PAH vs. LVSD was based on Mann–Whitney *U* test (*p* < 0.05)

**Table 2:** Hemodynamic parameters of in PAH patients

Parameter	*n*	Median (IQR)
SAP (mmHg)	19	133.0 (16.0)
mPAP (mmHg)	19	39.0 (19.0)
mPAWP (mmHg)	19	6.0 (6.0)
mRAP (mmHg)	19	7.0 (5.0)
CO (l × min^−1^)	19	4.0 (1.8)
CI (l × min^−1^ × m^−2^)	18	2.3 (0.5)
PVR (WU)	18	7.7 (5.7)
SVR (WU)	18	21.0 (8.7)
PVR/SVR	17	0.4 (0.2)
PA sat (%)	19	60.3 (9.3)
Art sat (%)	18	89.4 (5.0)

Data are presented as median (interquartile range)

*SAP* systemic arterial pressure, *PAP* pulmonary artery pressure, *PAWP* pulmonary artery wedge pressure, *CO* cardiac output, *CI* cardiac index, *PVR* pulmonary vascular resistance, *WU* wood units, *SVR* systemic vascular resistance, *PA* pulmonary artery, *sat* saturation

### Plasma ADMA, SDMA, and l-arginine concentrations

The plasma ADMA, SDMA, and l-arginine levels in PAH, LVSD, and healthy subjects are shown in Table 3. In PAH, median ADMA was 0.50 (0.34–0.91) μM, SDMA 0.83 (0.47–2.43) μM, l-arginine 55.1 (30.0–116.0) μM, l-citrulline 23.5 (10.6–45.1) μM, l-ornithine 83.2 (33.4–126.7) μM, l-arginine/ADMA ratio 102.2 (59.3–230.2), l-arginine/l-ornithine ratio 0.72 (0.35–1.36), and l-arginine/(l-ornithine + l-citrulline) ratio (GABR) 0.51 (0.28–1.08).

**Table 3:** Plasma ADMA, SDMA, and l-arginine levels in PAH, LVSD, and healthy subjects

Parameter	PAH		LVSD		Healthy subjects	
	*n*	median (IQR)	*n*	median (IQR)	*n*	median (IQR)
ADMA (µM)	21	0.50 (0.26)	14	0.56 (0.11)	27	0.36 (0.09)*
SDMA (µM)	21	0.83 (0.31)	14	0.74 (0.34)	27	0.42 (0.07)*
l-Arginine (µM)	21	55.1 (21.4)	14	81.8 (18.3)*	27	85.8 (27.0)*
l-Arginine/ADMA	21	102.2 (52.5)	14	140.4 (32.2)	27	237.8 (84.1)*

Data are presented as median (IQR)

*ADMA* asymmetric dimethylarginine, *SDMA* symmetric dimethylarginine

* *p* value for comparison between PAH vs. LVSD and PAH vs. healthy subjects was based on Mann–Whitney *U* test (*p* < 0.05)

**Fig. 2:**
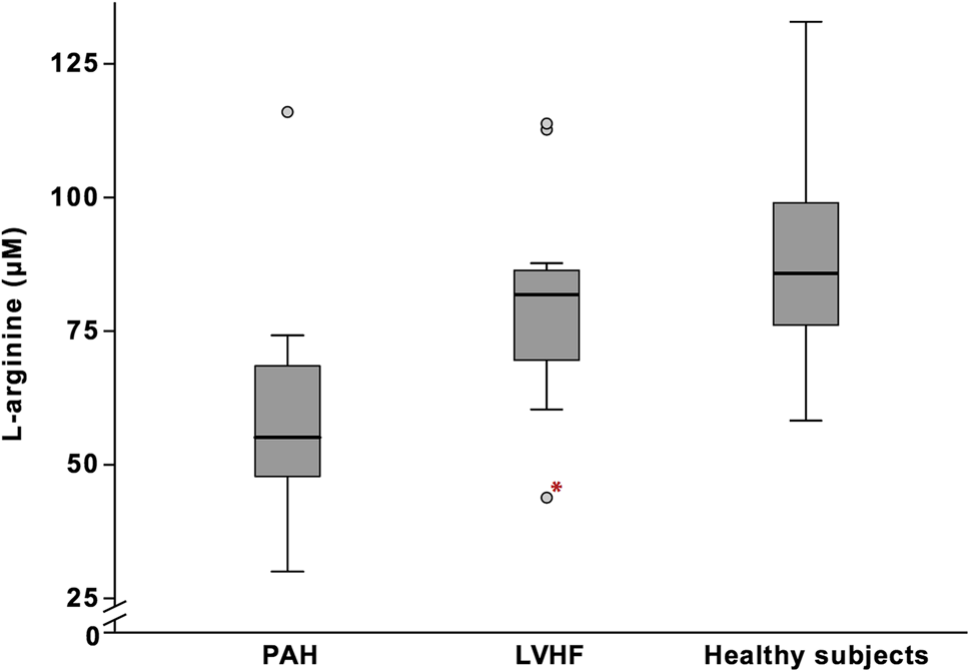
Plasma l-arginine levels in PAH, LVSD, and healthy subjects. *The marked outlier is the patient with PH due to left heart disease. There was a statistical significance between PAH vs. LVSD (*p* = 0.0012) and PAH vs. healthy subjects (*p* < 0.001), based on Mann–Whitney *U* test

In LVSD, median ADMA was 0.56 (0.50–0.74) μM, SDMA 0.74 (0.50–1.08) μM, l-arginine 81.8 (43.8–113.8) μM, and l-arginine/ADMA ratio 140.4 (81.2–214.8). The patient with PH with left heart disease had the lowest l-arginine levels (43.8 μM) in the LVSD group (Fig. 2). In healthy subjects, median ADMA was 0.36 (0.23–0.44) μM, SDMA 0.42 (0.32–0.59) μM, l-arginine 85.8 (58.2–132.8) μM, and l-arginine/ADMA ratio 237.8 (176.5–365.7).

ADMA and SDMA levels were significantly higher, and l-arginine levels and l-arginine/ADMA ratio were significantly lower in PAH compared to healthy controls (*p* < 0.001). Furthermore, patients with PAH had significantly lower l-arginine levels than patients with LVSD (*p* = 0.0012) (Fig. 2).

### Clinical determinants in relation to ADMA, SDMA, and l-arginine concentrations

**Fig. 3:**
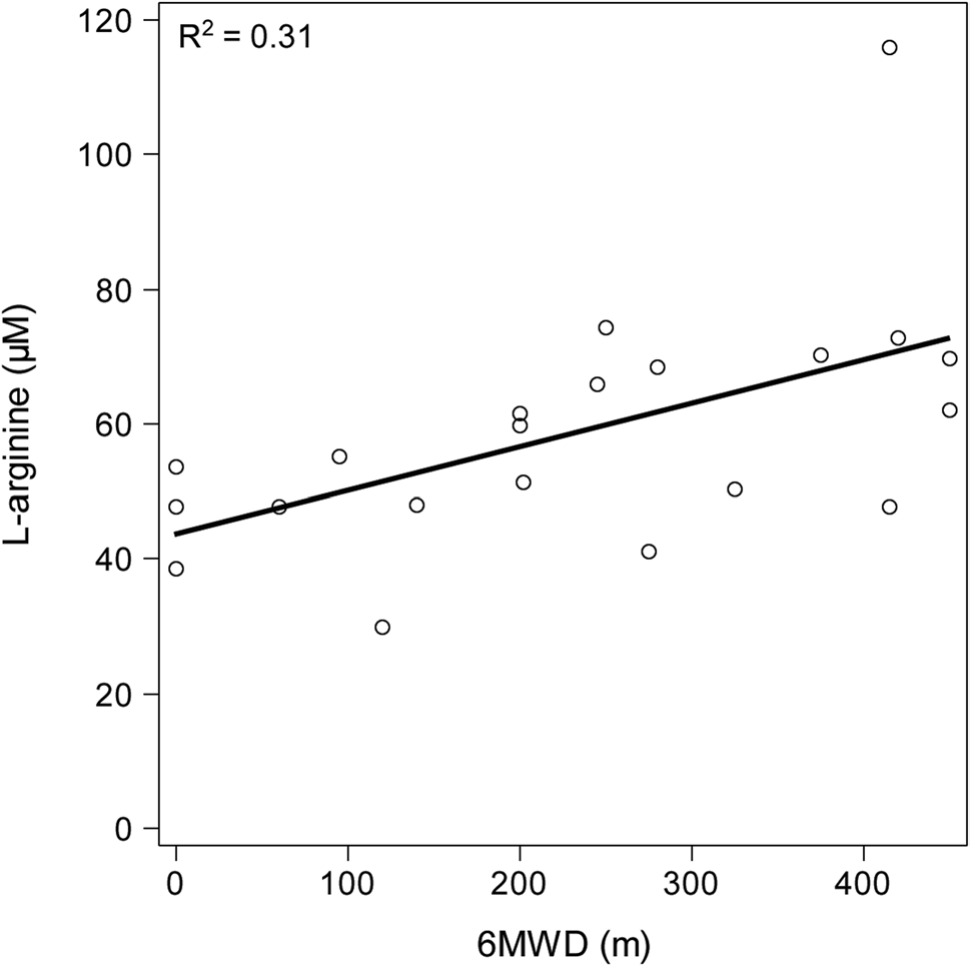
Association between 6MWD and l-arginine in 21 patients with PAH. Multiple regression analysis was used for statistical analysis

A Spearman’s correlation to determine the relationship between the clinical determinants, and l-arginine and dimethylarginines was performed. In PAH, l-arginine correlated positively with 6MWD (*r*_s_ = 0.58, *p* = 0.006) (Fig. 3) and correlated inversely with creatinine clearance (eGFR) (*r*_s_ = −0.52, *p* = 0.015). l-Arginine/ADMA ratio correlated inversely with WHO functional class (*r*_s_ = −0.46, *p* = 0.043) and eGFR (*r*_*s*_ = −0.49, *p* = 0.023). l-Ornithine correlated positively with 6MWD (*r*_s_ = 0.54, *p* = 0.012) and l-citrulline correlated inversely with eGFR (*r*_s_ = −0.44, *p* = 0.044). No significant correlations for cardiovascular risk factors or other clinical determinants were found, and no significant correlations between NT-proBNP and the above clinical determinants in PAH patients were found.

In LVSD, ADMA correlated inversely with 6MWD (*r*_s_ = −0.77, *p* = 0.004). SDMA correlated positively with WHO functional class (*r*_s_ = 0.54, *p* = 0.048). l-Arginine and l-arginine/ADMA ratio correlated inversely with eGFR (*r*_s_ = −0.69, *p* = 0.010) and (*r*_s_ = −0.56, *p* = 0.046), respectively. As in PAH patients, no significant correlations were found between NT-proBNP and clinical determinants were found.

### Haemodynamics in relation to ADMA, SDMA, l-arginine, l-citrulline, and l-ornithine concentrations

A Spearman’s correlation to determine the relationship between the hemodynamics and the plasma ADMA, SDMA, and l-arginine levels in PAH was performed. l-arginine/l-ornithine ratio and l-arginine/(ADMA + l-ornithine) ratio correlated inversely with mean aorta pressure (*r*_s_ = −0.56, *p* = 0.016) and (*r*_s_ = −0.67, *p* = 0.002), respectively. No other statistically significant relationships with haemodynamic parameters were seen for the metabolic markers. For NT-proBNP, there was a positive correlation with sPAP (*r*_s_ = 0.53, *p* = 0.024) and PVR/SVR (*r*_s_ = 0.50, *p* = 0.047). No other statistically significant relationship for NT-proBNP was seen.

## Discussion

The present study is the first to investigate the plasma levels of l-arginine and dimethylarginines in PAH compared to LVSD. We found that treatment naïve PAH patients have significantly lower baseline l-arginine levels compared to patients with LVSD on standard medication. In addition, treatment naïve PAH patients have significantly higher plasma ADMA and SDMA and lower plasma l-arginine levels as well as lower l-arginine/ADMA ratios compared to healthy subjects. Furthermore, l-arginine and l-arginine/ADMA ratio correlated to 6MWD and WHO functional class, respectively, in PAH patients. These results indicate that l-arginine levels may be able to distinguish PAH from LVSD and from healthy subjects.

Decreased l-arginine in PAH patients compared to patients with LVSD is an important finding in the present study. l-Arginine is not exclusively metabolized through NOS to NO and l-citrulline, as it may also be transformed to l-ornithine and urea by arginase [4]. Increased arginase activity leads to lower l-arginine levels and reduces NO production [25]. It is known that PAH patients have higher arginase activity compared to healthy controls, leading to an increased breakdown of l-arginine and thus affects NO synthesis [26]. Furthermore, PAH patients have increased arginase expression in endothelial cells and produce lower levels of NO compared to control cells in vitro [27]. Furthermore, arginase inhibition in a rat model and mice leads to reduced right ventricle systolic pressure and lung tissue remodeling as well as improved NO bioavailability, respectively, representing a potential treatment strategy in PH [28, 29]. We observed significantly decreased plasma l-arginine levels in PAH patients compared to both patients with LVSD and healthy subjects. The explanation to this may be related to the endothelial dysfunction in PAH in comparison to the heart involvement in LVSD. Therefore, the increased arginase activity in PAH, leading to lower l-arginine levels, could be one explanation to the difference in l-arginine between PAH and LVSD. In addition, the patient with PH due to left heart disease in the LVSD group had the lowest l-arginine levels. The increased portion of endothelial dysfunction in this patient may explain this. Our findings emphasize the potential value of l-arginine as a clinically relevant specific marker in PAH compared to LVSD. There are other disease states suggesting l-arginine as a potential marker [30, 31]; we believe that l-arginine may be a useful potential marker in many diseases that have impact on the NO system. Still, in our study, there was a significant difference in l-arginine between PAH and LVSD and this is an important finding. Furthermore, Beyer et al. showed a strong correlation between plasma l-arginine levels and severity of idiopathic PAH [32]. Currently, there are no known biomarkers to separate PAH to LVSD. Today, NT-proBNP is widely used in routine practice for PAH and LVSD [1]. Although NT-proBNP correlates with myocardial dysfunction and is elevated in all kinds of heart disease, it is not a specific marker for PH. Furthermore, l-arginine/ADMA ratio is considered to be a sensitive marker for atherosclerosis and an indicator of NOS function [33, 34]. In our study, l-arginine/ADMA ratio was decreased in both PAH and LVSD. However, we found a correlation between l-arginine and 6MWD and between the l-arginine/ADMA ratio and WHO functional class in patients with PAH. No correlation between l-arginine and dimethylarginines with 6MWD or NYHA functional class in LVSD was seen. Furthermore, there was no correlation between NT-proBNP and clinical determinants in PAH or LVSD. Today, the diagnostic work-up of PAH is time-consuming and expensive. l-Arginine as a potential marker for PAH might simplify this procedure in patients with suspected PAH.

PAH patients had significantly higher plasma levels of ADMA in comparison to healthy controls. Elevated ADMA levels may be the result of reduced renal elimination, inhibition by the metabolizing enzyme DDAH, or increased protein catabolism of PRMT (Fig. 1) [35]. The so-called PRMT–DDAH–ADMA pathway appears to play a crucially regulating role in the nitric oxide production [36]. Furthermore, the elevated ADMA levels enhance the inhibition of eNOS, which may lead to the uncoupling of eNOS, and result in a shift to production of superoxide’s instead of NO production [26]. Although not studied in this paper, S-nitrosylation products are also involved in the regulation of the above mentioned enzymes and thus availability of NOS substrates [37]. Plasma ADMA concentration was higher in both patients with PAH and LVSD as compared to healthy subjects in our study. Thus, elevated plasma ADMA levels should be considered a general marker of cardiovascular dysfunction rather than a specific marker of PAH.

Of note, we also found significantly higher plasma SDMA levels in PAH and LVSD patients compared to healthy controls. So far, SDMA has received only limited interest in PAH diagnostics, probably due to lack of evidence for a direct interference of SDMA with NOS activity [38]. However, SDMA may be indirectly involved by competing with l-arginine for the human cationic amino transporter (CAT) hCAT-2B, thereby hampering transport of l-arginine into endothelial cells [5, 7]. Thus, SDMA could indirectly impair NO production by reducing intracellular l-arginine supply [5]. Elevated plasma SDMA levels have been described in patients with IPAH and PAH due to sickle cell disease [5, 39]. In an earlier study of the acute effects of vardenafil in PH patients, ADMA and SDMA showed a positive correlation with baseline mRAP, indicating a more severe pulmonary vascular disease [23]. Surprisingly, no correlations between l-arginine, ADMA, SDMA, l-citrulline or l-ornithine, and haemodynamic parameters in patients with PAH were seen in this study. Still, SDMA is more closely correlated with renal function than ADMA [9, 38] and is not, as opposed to ADMA, a substrate for DDAH [40]. Actually, SDMA has been considered as an endogenous marker of eGFR [41].

Our study contains several limitations that need to be addressed. The number of patients included was limited, as PAH is a rare disease making it difficult to recruit a larger number of treatment naïve patients within a reasonable time frame. Furthermore, concomitant diseases and drug treatment for these diseases, such as diabetes mellitus, hypercholesterolemia, and hypertension, might have confounded our findings on l-arginine and other dimethylarginines. Still, this is the first study to compare baseline levels of l-arginine and dimethylarginines between PAH, LVSD and healthy subjects. Further studies in larger materials are warranted to confirm our findings concerning l-arginine as a potential marker for PAH.

## Conclusion

In conclusion, treatment naïve PAH patients had lower l-arginine levels than patients with LVSD. Moreover, l-arginine and the l-arginine/ADMA ratios were lower, while ADMA and SDMA levels were higher in PAH patients compared to healthy subjects. Furthermore, l-arginine correlated with 6MWD and the l-arginine/ADMA ratio correlated to WHO functional class in PAH. Thus, l-arginine may be a useful potential marker for differentiation between PAH and LVSD.
